# Review of "*Mosquito Net: A story of the pioneers of tropical medicine*" by Cyril Fox

**DOI:** 10.1186/1756-3305-2-2

**Published:** 2009-01-05

**Authors:** Bart GJ Knols

**Affiliations:** 1 Laboratory of Entomology, Wageningen University and Research Centre, P.O. Box 8031, 6700 EH, Wageningen, The Netherlands

## Review

*Mosquito Net *starts in more than one interesting way. The title suggests a story on bednets, but there is little to be read on these. The cover has an image of a dragon fly; one would have expected a mosquito, or perhaps portraits of the four men the book focuses on: Manson, Ross, Reed, and Gorgas. And it starts with a visit, in 1920, of King George V to the hospitalized William Gorgas a few days before his death following a stroke. What follows are 19 chapters of intriguing, sometimes gripping, descriptions of events that led these four men to great discoveries and victory over mosquito-borne diseases based on the scientific knowledge they generated. It is also a story of hardship, perseverance, eureka moments, fame, Nobel Prizes, bitterness, and rivalry that will be fascinating to the novice and, alas, *déjà vu *to the expert reader.

When Manson, as newly graduated medical officer arrived in China, he met hostile communities not willing to accept the little western medicine had to offer at the time. Missionaries despised him for not spreading the gospel and showing more interest in the 'new' diseases he encountered, notably elephantiasis. Void of a microscope and clues on the aetiology of the disease, it was not until Manson returned to London in 1875 that he learnt about filarial worms (now known as *Wuchereria bancrofti*) causing the symptoms he observed in Chinese patients. Virtually nothing was known about mosquitoes in those days, apart from the nuisance they cause. He became intrigued by filarial worm passage between humans and was the first to dissect mosquitoes and find live parasites in their gut. Mistakes were made. Manson thought that it was not the mosquito bite that introduced microfilariae, but that human consumption of water in which the mosquitoes with worms had died after egg laying completed their life-cycle. For years, together with his discovery that microfilariae in the human host migrate to the peripheral blood at night, this theory was too much for the establishment back home to accept. He faced skepticism and was ridiculed by his peers, indeed a fate that accompanies most major breakthroughs even today. It befell Low, in the early 1900s, to demonstrate that the bite of an infected mosquito introduced microfilariae in humans. After several decades of hardship in China, Manson was only to embark on malaria as the second disease for which he hypothesised a role for mosquitoes in its transmission after returning to London in 1890. In fact, it was not until he met with another Scotsman, Ronald Ross, in 1894, that the transmission cycle of malaria started to become unfold.

Ross served the Indian Medical Service as of 1881 as a regular surgeon, but developed an interest in elucidating the causes of disease rather than just treating them. Although discouraged by his superiors, and later on severely ridiculed by them, Manson challenged Ross with a simple question that led him to spend hundreds of hours staring through a primitive microscope: "*Follow the flagellum"*. In 1880, Laveran, a French surgeon serving in Algeria, was the first to observe exflagellation of the male gametocyte, and Manson was convinced that these flagella played some role in parasite transmission. Armed with this knowledge, Ross returned to India, only to be faced with the problem of recruiting volunteers for his experiments. This made him turn to bird malaria – a choice that he, no doubt, was to regret later in life. Thousands of miles away, in London, Manson mentored and encouraged Ross and shielded him from the politics surrounding their endeavour; the two exchanged some 165 letters within a few years. When Ross observed the more elusive 'dappled winged' *Anopheles *mosquitoes, and started including these in his experiments, it was only a matter of time before he was to observe parasite development in the mosquito. This day was 20 August 1897, since known as 'Mosquito Day'. In a state of euphoria Ross wrote to Manson, only to be transferred the following day to Kherwara, a location without malaria. Raged with anger against his superiors, Ross had to wait for nearly a year, after his next transfer to Calcutta, to demonstrate the presence of thread-like parasites (sporozoites) in the salivary glands of anophelines. Manson immediately reported these findings to the British Medical Association, in July 1898, causing a great sensation – it earned Ross a Nobel Prize in 1902.

The now famous Ross, however, got entwined for the rest of his life in a battle with all around him (including Manson) as to being the first to nail the mosquito-malaria theory. He turned increasingly sour, fuelled by the claims of Italian researchers (notably Grassi) who completed the malaria life cycle in humans (not birds) at around the same time as Ross. Fox devotes a 25 page chapter, titled 'Personality', to Ross' frustration and anger towards others progressing the science of malaria. Ross published '*The Prevention of Malaria*' in 1911, and became a zealous advocate of malaria eradication based on mosquito control. Much of what was stated therein still holds today, and should perhaps be mandatory literature for those now entering the second, recently launched, era of malaria eradication.

Across the Atlantic, Ross' discoveries were taken much more seriously in 1900, at the time when the US surgeon-general ordered a Commission, headed by Walter Reed, to discover the cause of yellow fever in Havana, Cuba. Reed, a medical doctor and bacteriologist, quickly learnt that Ross' approach did not work for yellow fever as there was simply nothing to observe through the microscope (not surprisingly, as yellow fever is of viral origin). Instead, they focused on the way the disease is spread. Although mosquitoes were suspected to play a role as early as 1881 (as persistently claimed by the Cuban doctor Carlos Finlay) the 'fomites theory' persisted too, which claimed that yellow fever spread through contact with patients, their clothing, and bodily fluids. This theory was only falsified after three non-immune volunteers were locked up for twenty nights in an experimental hut with bedding and clothing from deceased yellow fever patients and not developed any symptoms themselves. Next they partitioned the hut, with volunteers in both compartments, of which one had mosquitoes that previously gorged on yellow fever patients. At risk of loosing their lives, a third of those in the mosquito compartment fell ill; luckily, all survived. Within three weeks after starting the hut trials Reed and his group had cracked the mosquito theory.

The latter part of the book focuses on the appropriation of this new knowledge, first in Havana, and later during the construction of the Panama Canal. Fox then introduces the last of the four pioneers: William Gorgas. He arrived as Chief Sanitary Officer in Havana in 1898. When he learnt of the Reed Commission's discoveries he staged one of the first urban vector control programmes in history through larviciding (with kerosene) and screening of houses. Whereas 300 souls succumbed to yellow fever in 1900, by the end of 1901 the disease was eliminated. In Panama, under the command of De Lesseps, the French had commenced the construction of the Canal in 1879, but abandoned the project within a decade – a third of the French workforce had died because of malaria and yellow fever that riddled the jungle. The Americans took over in 1904 after having been granted the perpetuity of use, occupation and control over the Canal zone which lasted until the turn of the millennium. A decade later the Canal was completed, with Gorgas' sanitation efforts having saved countless lives of the indigenous and US work forces. Gorgas used the hand of the law that prohibited the presence of larvae within distance of human habitation and staged a massive mosquito control campaign. Several thousand sanitation workers reduced mosquito breeding and screened thousands of houses along the 50 mile stretch of the Canal. By 1906 yellow fever had disappeared, and malaria cases dropped from 821/1000 in that year to 70/1000 by 1913 – the Canal was opened in August 1914, on the day WWI ignited in Europe. A year later, Gorgas was promoted to Major-General by special order of Congress, the highest rank ever achieved by a doctor in the US Army.

Although the contributions of these men laid the foundations for tropical medicine as we know it today, Fox does recognize their paternalistic and imperialistic motives fuelled during that era. However, and rightly so, their relentless efforts also introduced the shift towards universal medicine, as opposed to colonial medicine. Fox's book may be seen as another historic account of fascinating discoveries. Yet, anyone in tropical medicine today, novice or expert, will be left with one clear message after reading it. Great discoveries are still left to be made, and even poorly equipped field laboratories in developing countries can make these. Above all, this book tells us that passion, motivation and drive, and a keen scientific eye are no less important today than in the early days of tropical medicine – hence, a very worthy and stimulating read.

## Competing interests

The author declares that they have no competing interests.

